# A Human Renal Proximal Tubule Cell Line with Stable Organic Anion Transporter 1 and 3 Expression Predictive for Antiviral-Induced Toxicity

**DOI:** 10.1208/s12248-016-9871-8

**Published:** 2016-01-28

**Authors:** Tom T. G. Nieskens, Janny G. P. Peters, Marieke J. Schreurs, Niels Smits, Rob Woestenenk, Katja Jansen, Thom K. van der Made, Melanie Röring, Constanze Hilgendorf, Martijn J Wilmer, Rosalinde Masereeuw

**Affiliations:** Department of Pharmacology and Toxicology, Radboud Institute of Molecular Life Sciences, Radboud University medical center, Nijmegen, The Netherlands; Department of Laboratory Medicine - Laboratory of Hematology, Radboud University Medical Centre, Nijmegen, The Netherlands; Innovative Medicines, Drug Safety and Metabolism, AstraZeneca R&D, Mölndal, Sweden; Division Pharmacology, Utrecht Institute for Pharmaceutical Sciences, Utrecht University, Utrecht, The Netherlands; Department of Pharmacology and Toxicology, Radboud Institute for Molecular Life Sciences, Radboud University medical centre, P.O. box 9101, 6500 HB Nijmegen, The Netherlands

**Keywords:** antivirals, drug-drug interactions, nephrotoxicity, organic anion transport, proximal tubule epithelial cell

## Abstract

Drug-induced nephrotoxicity still hampers drug development, because current translation from *in vitro* or animal studies to human lacks high predictivity. Often, renal adverse effects are recognized only during clinical stages of drug development. The current study aimed to establish a robust and a more complete human cell model suitable for screening of drug-related interactions and nephrotoxicity. In addition to endogenously expressed renal organic cation transporters and efflux transporters, conditionally immortalized proximal tubule epithelial cells (ciPTEC) were completed by transduction of cells with the organic anion transporter (OAT) 1 or OAT3. Fluorescence-activated cell sorting upon exposure to the OAT substrate fluorescein successfully enriched transduced cells. A panel of organic anions was screened for drug-interactions in ciPTEC-OAT1 and ciPTEC-OAT3. The cytotoxic response to the drug-interactions with antivirals was further examined by cell viability assays. Upon subcloning, concentration-dependent fluorescein uptake was found with a higher affinity for ciPTEC-OAT1 (K_m_ = 0.8 ± 0.1 μM) than ciPTEC-OAT3 (K_m_ = 3.7 ± 0.5 μM). Co-exposure to known OAT1 and/or OAT3 substrates (viz. para-aminohippurate, estrone sulfate, probenecid, furosemide, diclofenac, and cimetidine) in cultures spanning 29 passage numbers revealed relevant inhibitory potencies, confirming the robustness of our model for drug-drug interactions studies. Functional OAT1 was directly responsible for cytotoxicity of adefovir, cidofovir, and tenofovir, while a drug interaction with zidovudine was not associated with decreased cell viability. Our data demonstrate that human-derived ciPTEC-OAT1 and ciPTEC-OAT3 are promising platforms for highly predictive drug screening during early phases of drug development.

**Electronic supplementary material:**

The online version of this article (doi:10.1208/s12248-016-9871-8) contains supplementary material, which is available to authorized users.

## INTRODUCTION

The renal proximal tubules play a major role in eliminating waste products from the body, including drugs and their metabolites. Their active secretion and reabsorption mechanisms together with biotransformation capacity make proximal tubule cells especially sensitive to drug-induced toxicity and subsequent acute kidney injury (AKI) (1). Not surprisingly, nephrotoxicity is a significant cause for drug attrition during pharmaceutical development, often recognized only during clinical stages of development as translation from *in vitro* and animal studies to human lacks high predictivity (2,3).

An in vitro model with high predictive value for drug-induced nephrotoxicity should closely reflect the *in vivo* processes involved in renal drug handling. More specific, a robust cell-based model should include a proximal tubule epithelium stably expressing a broad range of functional transporters and metabolic enzymes that act in concert in renal drug elimination (4). This process may be affected in concomitant drug treatment, leading to clinically relevant drug-drug interactions (DDI). The renal elimination mechanism of xenobiotics can roughly be divided into two major pathways, viz. the organic anion and the organic cation system. As a first step in elimination of organic anions in humans, active tubular uptake is mediated by the organic anion transporter 1 (OAT1; *SLC22A6*) and organic anion transporter 3 (OAT3; *SLC22A8*) present at the, blood-facing, basolateral side (5). These transporters are characterized by their high affinity and capacity and, as a consequence, are major players in the development of drug-induced nephrotoxicity (6). After uptake, secretion of anionic compounds into the tubular lumen is facilitated by apically expressed efflux transporters, such as the multidrug resistance proteins 2 and 4 (MRP2 and -4; *ABCC2* and *-4*) and breast cancer resistance protein (BCRP; *ABCG2*) (7). In parallel, renal elimination of organic cations in the human proximal tubular epithelium is facilitated by basolateral uptake, predominantly via the organic cation transporter 2 (OCT2; *SLC22A2*), and apical efflux via multidrug and toxin extrusion proteins 1 and 2-K (MATE1 and -2-K; *SLC47A1* and *-2*) (8) and P-glycoprotein (P-gp; *ABCB1*) (9).

Renal drug transporters demonstrate a large overlap in substrate specificity, introducing redundancy in uptake mechanisms of proximal tubule cells, and contributing to the relative high sensitivity of the tissue (6,10). This especially counts for organic anions, as this class comprises the majority of drugs that are excreted by the kidneys. Drug-induced nephrotoxicity related to the proximal tubular epithelium by this class of compounds have been described broadly, including for the acyclic nucleotide phosphonates adefovir, cidofovir, and tenofovir (11,12). These antiretroviral compounds are used for treatment of HIV, hepatitis B, and cytomegalovirus infections and function as nucleotide analog reverse transcriptase inhibitors (NtRTIs) (13). The exact mechanism of antiviral-induced renal toxicity is still under debate (14), but the involvement of OATs in the uptake of many antivirals has been widely acknowledged (15–17). To prevent NtRTI-induced nephrotoxicity, their uptake can be inhibited by co-administration of an OAT1 inhibitor, such as probenecid (18). As with many other diseases, current antiviral therapy in HIV infections is based on polypharmacy. Increased plasma concentrations and systemic toxicity have been observed with didanosine co-administration of tenofovir in anti-HIV triple therapy, possibly by DDI at the site of OAT1 that limited renal excretion (19). Together, polypharmacy can optimize the life-span of infected patients, but this strategy simultaneously increases the risk for DDI and demands for personalized evaluation of the benefit/risk ratio for each drug (20).

The aim of this study was to establish a robust human cell model that allows prediction of drug-induced nephrotoxicity and DDI of organic anions, with a focus on antivirals. We evaluated conditionally immortalized proximal tubule epithelial cells (ciPTEC) as a preclinical in vitro prediction model (21). This model already demonstrated to be highly predictive for studying DDI at the site of OCT2 (22) and to endogenously exhibit metabolic enzymes (23) together with a panel of functional efflux transporters (21,24). However, the expression of OAT1 and OAT3 was rapidly lost in culture. Here, these transporters were stably expressed in ciPTEC by transduction, followed by an elegant selection procedure using OAT transporter functionality, completing the relevant renal xenobiotic transporters in ciPTEC. The function of both transporters appeared to be stable upon prolonged culturing. These unique characteristics of the presented OAT containing human cell lines allowed screening for DDI using known pharmacological OAT1 and OAT3 substrates and/or inhibitors. Upon validation, we demonstrated that OAT-mediated uptake in ciPTEC are key determinants in antiviral-induced cytotoxicity. These findings underscore that ciPTEC-OAT1 and ciPTEC-OAT3 are valuable tools for drug-induced toxicity screening.

## MATERIALS AND METHODS

### Cell Culture

Conditionally immortalized proximal tubule epithelial cells (ciPTEC) were developed as described by Wilmer *et al.* with informed consent of the donors in accordance with the approved guidelines of the Radboud Institutional Review Board (21). Cells were seeded 7 days prior to the experiment at their corresponding density (55,000 cells/cm^2^ for ciPTEC parent cells, 63,000 cells/cm^2^ for ciPTEC-OAT1, and 82,000 cells/cm^2^ for ciPTEC-OAT3) and grown for 1 day at 33°C and 5% *v/v* CO_2_ to allow proliferation, enabled by the temperature-sensitive mutant of SV large T antigen (SV40T). Next, cells were cultured for 6 days at 37°C and 5% *v/v* CO_2_ to stimulate differentiation and formation of an epithelial monolayer, described as “maturation.” Cells were cultured using Dulbecco’s modified eagle medium (DMEM HAM’s F12, Life Technologies, Paisly, UK), 5 μg/ml insulin, 5 μg/ml transferrin, 5 μg/ml selenium, 35 ng/ml hydrocortisone, 10 ng/ml epidermal growth factor (EGF), 40 pg/ml tri-iodothyronine (Sigma, St. Louis, USA), and 10% fetal calf serum (FCS, Greiner Bio One, Kremsmuenster, Austria). Medium was refreshed every second day, supplemented with 1% penicillin/streptomycin (pen/strep, Invitrogen, Carlsbad, USA) at 33°C and without pen/strep at the maturation temperature of 37°C. Three T3 mouse-fibroblast (3 T3) cells were cultured at 37°C and used only as irradiated non-proliferating feeder cells for sub-cloning procedures upon transduction, as described (21).

### Vector Construction

Vector construction was performed using Gateway Cloning Technology (Invitrogen), according to the manufacturer’s instructions. Commercially obtained vectors containing OAT1 (pENTR201-hOAT1, Harvard Plasmids HsCD00044153) and OAT3 (pENTR201-hOAT3, HsCD00044090) were transferred into a pLenti4/V5-DEST vector by LR recombinant reaction, resulting in expression vectors pLenti4/V5-EX-hOAT1 and pLenti4/V5-EX-hOAT3. The inducible CMV-TetO2 promoter was replicated from pcDNA5-FRT-TO (Invitrogen) using primers that introduce ClaI (forward Cla1-CMV-TetO2: GCCGCCATCGATGCCGCCGTTGACATTGATTATTGACT) and EcoRI restriction sites (reverse EcoRI-CMV-TetO2: GGCGGCGAATTCGGCGGCCGGAGGCTGGATCGGTCCCGG). The resulting PCR product (ClaI-CMV-TetO2-EcoRI) was purified using the High Pure PCR Product Purification kit (Roche, Basel, Switzerland). Both PCR product and expression vectors were digested by ClaI and EcoRI (New England Biolabs, Ipswich, USA) for 1 h at 37°C and, after purification, ligation was performed with a 1:3 (insert:vector) unit ratio using T4 ligase (Invitrogen) for 2 h at 37°C, resulting in the pLenti expression constructs (pLenti4/V5-EX-CMV-TetO2-hOAT1 and pLenti4/V5-EX-CMV-TetO2-hOAT3).

### OAT Transduction in ciPTEC

To obtain lentiviral particles containing the OAT constructs, lentiviral stock was produced by transfecting the pLenti expression constructs with packaging plasmid mix into the HEK293FT cell line using ViraPower Lentiviral Gateway Expression Systems (Invitrogen), according to the manufacturer’s instructions. CiPTEC were cultured to 50–70% confluency and exposed to lentiviral particles for 24 h. Both ciPTEC-OAT1 and ciPTEC-OAT3 were selected and subcloned to obtain a homogeneous cell population. To this end, transduced ciPTEC-OAT3 cells were plated into three separate culture flasks (100, 300, and 900 cells) containing irradiated (30 Gy) non-proliferating 3 T3 cells as described by Saleem *et al*. (25). After 2–3 weeks, single cell colonies of ciPTEC-OAT3 were picked and cultured. Transduction efficiency for ciPTEC-OAT1 was lower than for ciPTEC-OAT3, making immediate subcloning difficult. Therefore, the heterogeneous cell population of ciPTEC-OAT1 was enriched by positive selection of fluorescein-transporting cells. Only successfully transduced ciPTEC express functional OAT; hence, positive selection could be performed upon exposure to the OAT substrate fluorescein using BD FACSAria SORP flow cytometer (BD biosciences, San Jose, USA). Twenty million ciPTEC-OAT1 cells were suspended in HBSS (Invitrogen) containing 1 μM fluorescein and incubated for 10 min at 37°C before fluorescence-activated cell sorting (FACS). Enriched ciPTEC-OAT1 cells were subcloned as described for ciPTEC-OAT3. Both ciPTEC-OAT1 and ciPTEC-OAT3 were cultured for up to 30 passages after transduction to study stability of OAT1 and OAT3 expression.

### OAT-Mediated Fluorescein Uptake

To evaluate OAT transporter function and inhibition properties of several known OAT substrates, fluorescein uptake was measured by flow cytometry and multi-plate reader. Mature monolayers of sub-cloned ciPTEC spanning 29 passages were co-incubated with fluorescein (1 μM, unless stated otherwise) and a test compound in HBSS for 10 min at 37°C. Compounds known for their inhibitory effect on OAT-mediated transport, para-aminohippuric acid (PAH), estrone sulfate, probenecid, furosemide, cimetidine, diclofenac, adefovir, cidofovir, tenofovir, and zidovudine, were tested. The organic cation metformin was included as a negative control. All chemicals were obtained from Sigma, unless stated otherwise. Uptake was stopped by washing three times with ice-cold HBSS (4°C). For flow cytometry, samples were harvested following fluorescein exposure using trypsin-EDTA, washed, fixed using 0.5% paraformaldehyde, and measured using FACS calibur (Becton Dickinson, Franklin Lakes, USA). For 96-well plate assay, cells were lysed by 200 μl 0.1 M NaOH for 10 min at 37°C, and fluorescence was measured (exCitation 485 nm, emission 535 nm) using the multiplate reader Victor X3 (Perkin Elmer, Waltham, USA).

### Viability Assays

To evaluate toxicity induced by antivirals, viability of ciPTEC was evaluated by an MTT assay (26). Briefly, monolayers of ciPTEC (96-wells) were exposed to antivirals in serum-free medium (SFM) on day 6 of maturation. Cell toxicity was analyzed further in presence of MRP and BCRP efflux inhibitors MK571 (5 μM) and KO143 (10 μM). After incubation for 24, 48, and 72 h at 37°C, ciPTEC were washed and incubated with 0.5 mg/ml thiazolyl blue tetrazolium bromide (MTT, Sigma) for 3 h at 37°C in absence of antivirals. Formazan crystals formed in viable cells were dissolved in dimethyl sulfoxide (DMSO, Merck, Whitehouse Station, USA), and optical density was measured (560 nm, background at 670 nm was subtracted) using Benchmark Plus (Bio-Rad, Hercules, USA).

### Gene Expressions in ciPTEC

Total RNA was isolated from matured ciPTEC (6-well plates) spanning 10 passages for ciPTEC-OAT1 and 11 passages for ciPTEC-OAT3 using TRIzol (Life Technologies Europe BV) and chloroform extraction. Complementary DNA (cDNA) was synthesized using M-MLV Reverse Transcriptase (Promega, Madison, USA), according to the manufacturer’s instructions. The messenger RNA (mRNA) expression levels were evaluated using gene-specific primer-probe sets obtained from Life Technologies: OAT1 (*SLC22A6*, hs00537914), OAT3 (*SLC22A8*, hs00188599), *GAPDH* (hs99999905), and TaqMan Universal PCR Master Mix (Applied Biosystems). The quantitative PCR reactions were performed using CFX96-Touch Real-Time PCR System (BioRad) and analyzed using BioRad CFX Manager (version 1.6). mRNA levels for ciPTEC-OAT1 and ciPTEC-OAT3 were calculated using GAPDH as a reference gene and compared to gene expressions in human kidney homogenates in triplicate.

### Data Analysis

A Michaelis-Menten equation was combined with linear diffusion to fit fluorescein uptake data after background subtraction with GraphPad Prism (version 5.03). For calculation of IC50 values, log (concentration inhibitor) *versus* fluorescein uptake was plotted after background subtraction using GraphPad Prism.

For MTT and fluorescein inhibition assays, data were normalized to the viability or activity of untreated control cells. Non-linear regression with variable slope constraining the top to 100% was used to fit the data after background subtraction with GraphPad Prism. Statistics was performed by two-way ANOVA (two-tailed, α = 0.05) using GraphPad Prism as well. All data is presented as mean ± SEM of at least three separate experiments (*n* = 3) performed in triplicate, unless stated otherwise.

## RESULTS

### Functional OAT Expression in ciPTEC

**Fig. 1:**
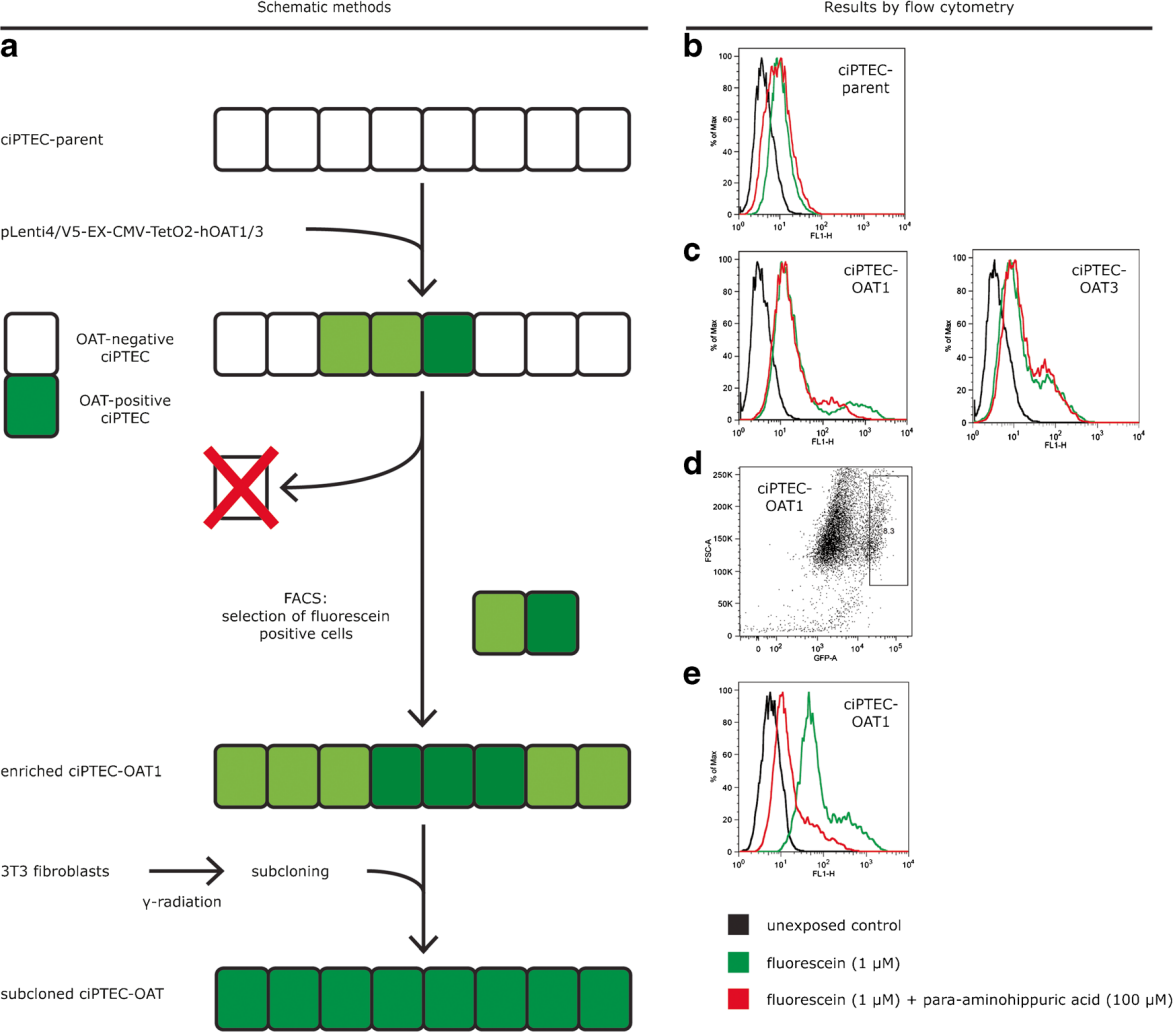
Schematic overview of transduction procedure to obtain ciPTEC-OAT1 and ciPTEC-OAT3. **a** CiPTEC parent was transduced with OAT1 or OAT3 lentiviral constructs and enriched by FACS using OATs’ capacity to transport fluorescein. Further subcloning using radiated 3 T3 fibroblasts as feeder cells resulted in a homogeneous ciPTEC-OAT1 or ciPTEC-OAT3 cell line. Histogram obtained by flow cytometry of **b** ciPTEC parent, **c** ciPTEC-OAT1, and ciPTEC-OAT3 exposed to fluorescein (1 μM, 10 min, *green line*), fluorescein and para-aminohippuric acid (100 μM, *red line*), or untreated cells (*black line*). Parent cells exposed to fluorescein did not show increased fluorescence intensity, while ciPTEC-OAT1 and ciPTEC-OAT3 both showed a sub-population with increased fluorescence indicative for OAT functionality, which is sensitive to para-aminohippuric acid-induce inhibition. **d** Scattered plot showing forward scatter (*y axis*) and fluorescein intensity (*x axis*) of transduced ciPTEC-OAT1 exposed to 1 μM fluorescein for 10 min. The population with high-fluorescence intensity indicated by *gate P1* (8.3% of total population) was sorted to enrich successfully transduced ciPTEC-OAT1. Transduction with OAT3 was more efficient than OAT1, represented by the larger positive subpopulation in Fig. 1c, making the enrichment protocol redundant for ciPTEC-OAT3. **e** Histogram of enriched ciPTEC-OAT1 exposed to fluorescein (1 μM, 10 min) in presence (*red line*) or absence (*green line*) of competitor para-aminohippuric acid (100 μM) demonstrates increased fluorescence intensity compare to non-enriched ciPTEC, but a heterogeneous population sensitive to para-aminohippuric acid, pointing towards the requirement of subcloning of the enriched cells

The absence of endogenous OAT1 and OAT3 expression in ciPTEC was demonstrated by exposure to fluorescein (1 μM) for 10 min, which did not increase the intracellular fluorescence intensity as measured by flow cytometry (Fig. 1b, red line). Therefore, OAT transporters were introduced separately by lentiviral transduction. A schematic overview of the experimental approach is provided in Fig. 1a. The transporter genes *SLC22A6* and *SLC22A8* were cloned under regulation of a CMV promoter and a TetO2 site to conditionally induce the expression. Remarkably, basal expression and function upon transduction of both OAT transporters was positive without tetracycline induction and was not influenced by this inducer (data not shown). Fluorescein uptake capacity (without induction by tetracycline) was used to discriminate between successfully transduced cells and non-transduced cells, reflected by a two sub-populations in the flow cytometer histogram (Fig. 1c). When exposed to 1 μM fluorescein for 10 min, a small cell population accumulated the fluorescent substrate, which was immediately selected using FACS. The fraction of OAT1-positive cells selected (Fig. 1d) accounted for only 8.3% of the total population. The enriched population accumulated fluorescein efficiently, and was sensitive to inhibition by para-aminohippuric acid, a known OAT1 substrates and/or inhibitors (Fig. 1e). The ciPTEC-OAT1 population enriched by FACS and the non-enriched ciPTEC-OAT3 population were subcloned to obtain homogeneous cell populations with high functional OAT transporter expression, demonstrated by qPCR. Expression levels of OAT1 and OAT3 in the respective cell lines were compared to gene expression levels in human kidney tissue homogenates, resulting in a ratio of 0.7 ± 0.2 and 0.14 ± 0.02 for OAT1 and OAT3, respectively. Intact tubular phenotype was demonstrated by functionally active OCT2, for which a drug interaction with cimetidine was shown to be similar to the parent cell line (Fig. S1).

### Drug-Interaction at the Site of OAT1 and OAT3

**Fig. 2:**
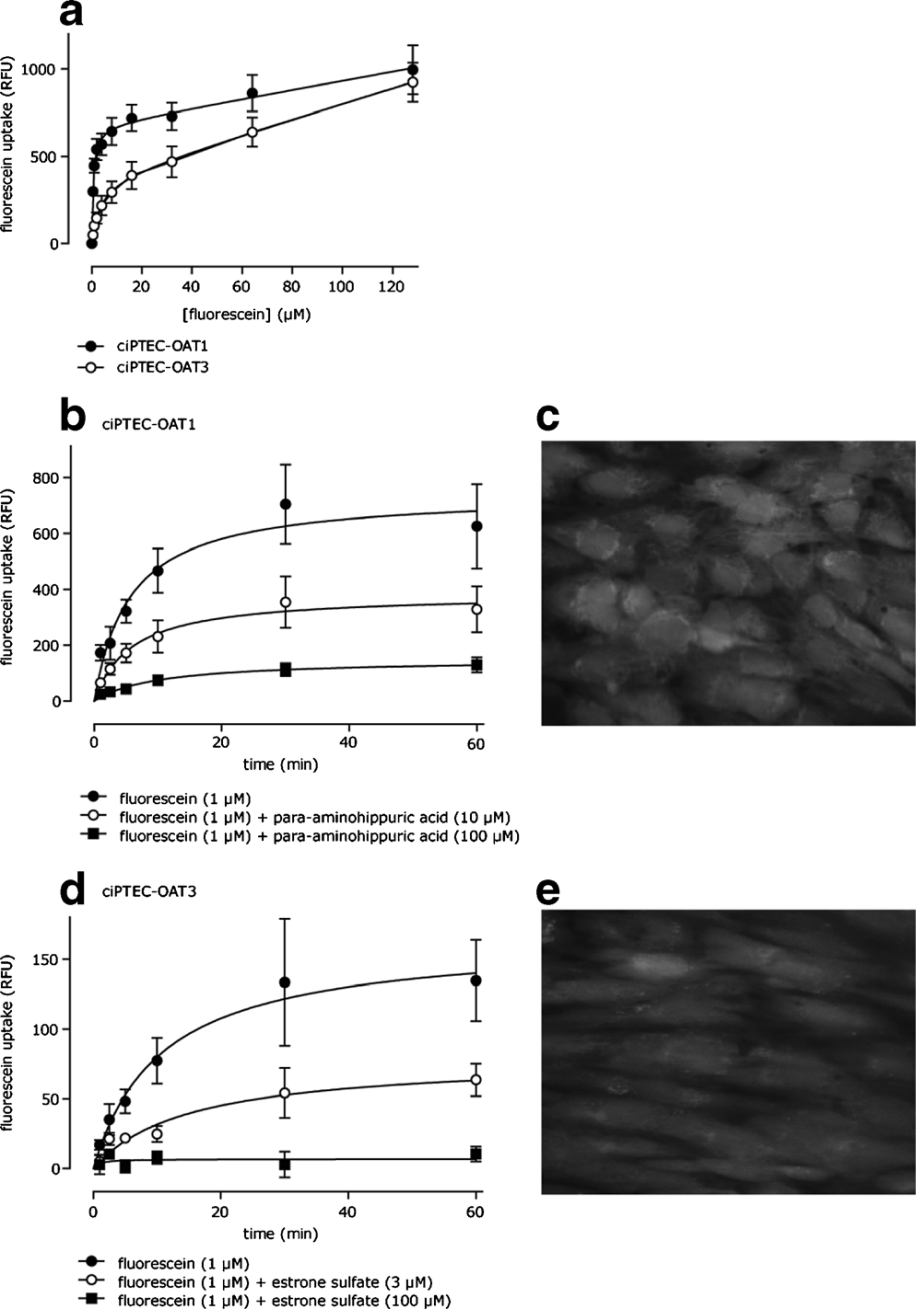
OAT-mediated fluorescein uptake in ciPTEC-OAT1 and ciPTEC-OAT3. **a** Concentration-dependent OAT1 and OAT3 mediated uptake of fluorescein after 10 min incubation in ciPTEC-OAT1 and ciPTEC-OAT3. The curve was fitted (*n* = 4) according to a Michaelis-Menten model in combination with linear diffusion. **b**, **c** Fluorescein uptake (1 μM) by ciPTEC-OAT1 and **d**, **e** ciPTEC-OAT3 up to 60 min in absence or presence of two concentrations of the typical inhibitors para-aminohippuric acid (PAH, for ciPTEC-OAT1) or estrone sulfate (ES, for ciPTEC-OAT3). **b**, **d** The curves were fitted (*n* = 4) to a standard saturation model after background subtraction. Analysis using two-way ANOVA indicated significantly decreased uptake curves in both ciPTEC-OAT1 (10 μM and 100 μM PAH, *p* < 0.001)) and ciPTEC-OAT3 (3 μM ES, *p* < 0.01; 100 μM ES, ***p < 0.001). **c**, **e** Representative images of fluorescein uptake (1 μM) by ciPTEC-OAT1 (**c**) and ciPTEC-OAT3 (**e**) after 10 min (magnification 20×)

**Fig. 3:**
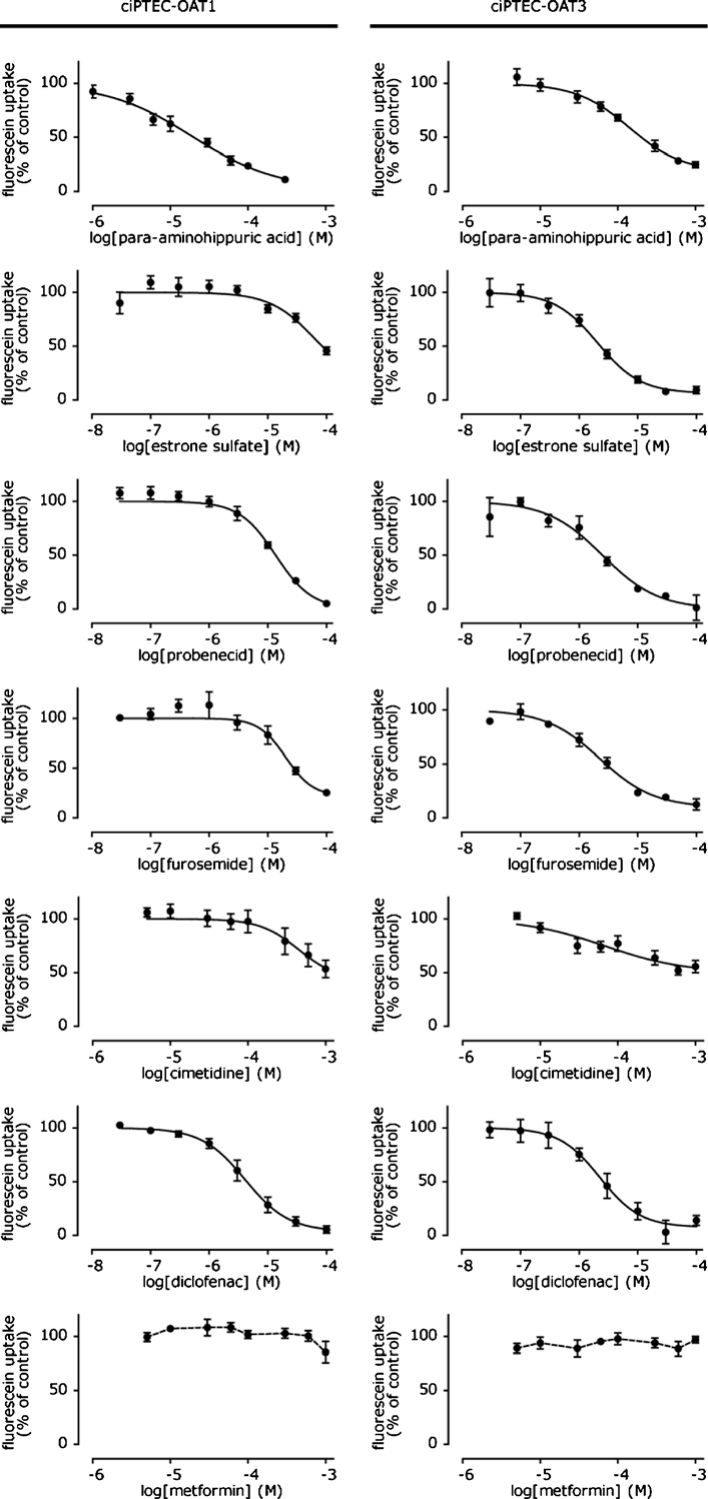
Inhibition of OAT-mediated fluorescein uptake by a panel of OAT-perpetrators. Fluorescein uptake (1 μM) by ciPTEC-OAT1 and ciPTEC-OAT3 when co-incubated with para-aminohippuric acid, estrone sulfate, probenecid, furosemide, cimetidine, diclofenac, and metformin for 10 min in HBSS at 37°C, relative to uptake without inhibitor. The *line* represents the fit according to a one-site competition model with variable slope, except for metformin. Values are derived from experiments performed at passage x + 8, x + 11, x + 14, and x + 29 upon transduction (*n* = 4)

Transport kinetics of OAT-mediated fluorescein transport was investigated further by studying the time and concentration dependent uptake of the substrate. Fluorescein uptake demonstrated partial saturation in OAT1- and OAT3-expressing cells (Fig. 2a, b, d) for which a K_m_ and V_max_ value was determined taking a passive diffusion component k_d_ into account (Table I). Fluorescein affinity was approximately fivefold higher for OAT1 than for OAT3. Upon fluorescein exposure (10 min, 1 μM), confocal fluorescent imaging confirmed uptake in ciPTEC-OAT1 and ciPTEC-OAT3 (Fig. 2c, e). To demonstrate the uptake was transporter mediated, specific inhibition of fluorescein uptake in presence of two concentrations para-aminohippuric acid (10 and 100 μM) and estrone sulfate (3 and 100 μM) in ciPTEC-OAT1 and ciPTEC-OAT3, was studied (Fig. 2b, d). CiPTEC-OAT1 and ciPTEC-OAT3 were evaluated further by determination of IC_50_ values using concentration-dependent inhibition of fluorescein uptake in presence of para-aminohippuric acid, estrone sulfate, probenecid, furosemide, cimetidine, and diclofenac (Fig. 3, Table II). Overall, IC_50_ values calculated in our models are in close agreement with previously reported values, although it should be noted that probe substrates may differ and influence IC_50_ values (Table II). Further confirmation of specificity was obtained using metformin, not affecting OAT-mediated fluorescein uptake in both ciPTEC-OAT1 and ciPTEC-OAT3, as metformin is an OCT substrate (38). The experiments depicted in Fig. 3 were performed in cells spanning 29 passages after transduction. The small variations in these data and maintained fluorescein uptake indicate stable transduction and high robustness of transporter function in ciPTEC-OAT1 and ciPTEC-OAT3.

**Table I:** Michaelis-Menten Parameters for OAT-Mediated Fluorescein Uptake in ciPTEC-OAT1 and ciPTEC-OAT3^a^

	ciPTEC-OAT1	ciPTEC-OAT3
K_m_ (μM)	0.8 ± 0.1	3.7 ± 0.5
V_max_ (RFU)	695 ± 84	384 ± 103
K_d_ (RFU*L/μmol)	2.4 ± 1.2	4.3 ± 0.9

^a^Data are expressed as mean ± SEM, *n* = 4

**Table II:** Inhibitory Potencies of Substrates and/or Inhibitors of Fluorescein Uptake in ciPTEC-OAT1 and ciPTEC-OAT3 and a Selection of Reference Values^a^

	Current study		Literature				
	Cell line	IC_50_ (μM)	IC_50_ (μM)	K_i_ (μM)	Substrate	Cell line	Ref
Para-aminohippuric acid	ciPTEC-OAT1	18 ± 4	8.8	6.02	6-carboxyfluorescein ochratoxin A	CHO-OAT1 S2-OAT1	(27,28)
	ciPTEC-OAT3	152 ± 3		19.6 100	ochratoxin A benzylpenicillin	S2-OAT3 HEK293-hOAT1	(28,29)
Estrone sulfate	ciPTEC-OAT1	54 ± 13	>100		PAH	S2-OAT1	(30)
	ciPTEC-OAT3	2.1 ± 0.3	3.0		estrone sulfate	Xenopus-OAT3	(31)
Probenecid	ciPTEC-OAT1	12.7 ± 0.5	6.3	4.29 12.1	ochratoxin A 6-carboxyfluorescein PAH	S2-OAT1 CHO-OAT1 S2-OAT1	(16,27,28)
	ciPTEC-OAT3	1.9 ± 0.6	3.1	4.41	cimetidine ochratoxin A	CHO-OAT3 S2-OAT3	(28,32)
Furosemide	ciPTEC-OAT1	25 ± 4	18		PAH	S2-OAT1	(33)
	ciPTEC-OAT3	2.3 ± 0.4	7.31 1.7		estrone sulfate sitagliptin	S2-OAT3 CHO-OAT3	(32,33)
Cimetidine^b^	ciPTEC-OAT1	654 ± 291	492		PAH	S2-OAT1	(34)
	ciPTEC-OAT3	215 ± 162	79 53		sitagliptin estrone sulfate	CHO-OAT3 Xenopus-OAT3	(32,35)
Diclofenac	ciPTEC-OAT1	5 ± 1	4.46 4		PAH adefovir	S2-OAT1 CHO-OAT1	(36,37)
	ciPTEC-OAT3	3 ± 1	7.78		estrone sulfate	S2-OAT3	(36)

^a^Data are expressed as mean ± SEM, n = 4

^b^Apparent IC_50_ value due to partial inhibition

### OATs Mediate Antiviral-Induced Toxicity

**Fig. 4:**
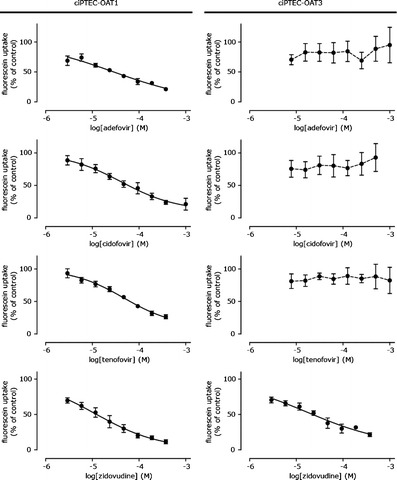
Inhibition of OAT-mediated fluorescein uptake by adefovir, cidofovir, tenofovir, and zidovudine. Fluorescein uptake (1 μM) by ciPTEC-OAT1 and ciPTEC-OAT3 when co-incubated with the antivirals for 10 min in HBSS at 37°C, relative to uptake without inhibitor. The *line* represents the fit according to a one-site competition model with variable slope (*n* = 4)

**Fig. 5:**
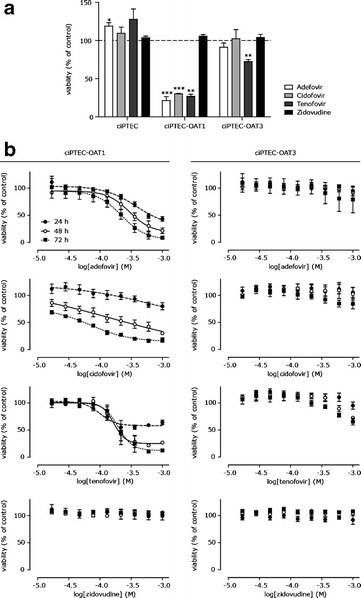
Antiviral-induced toxicity in ciPTEC-OAT1 and ciPTEC-OAT3. **a** Viability of ciPTEC parent, ciPTEC-OAT1, and ciPTEC-OAT3 after exposure to antiviral agent (1 mM) for 48 h in serum-free medium relative to cell viability as measured with the MTT assay without exposure (*n* = 3). **p < 0.01; ***p < 0.001. **b** Viability of ciPTEC-OAT1 and ciPTEC-OAT3 upon tenofovir, adefovir, cidofovir, or zidovudine exposure for 24, 48, and 72 h in serum-free medium, relative to cell viability without exposure. The *line* represents the fit according to a one-site competition model with variable slope (*n* ≥3)

As toxicity of antivirals was reported to be associated with OAT1- and OAT3-mediated renal tubular uptake, we investigated their effects on OAT function and cell viability upon drug exposures. Concentration-dependent inhibition of fluorescein uptake via OAT1 was observed by adefovir, cidofovir, tenofovir, and zidovudine, while OAT3 was only associated with zidovudine-fluorescein interactions (Fig. 4, Table III). Next, the DDI indices were determined. The US Food and Drug Administration (FDA) draft a DDI guideline (48) recommending to perform clinical DDI studies when the ratio between unbound plasma concentration and IC_50_ (C_max,u_ /IC_50_) is higher than 0.1. For adefovir, cidofovir, and zidovudine, the IC_50_ value was less than 10 times the maximal free plasma concentration (C_max,u_/IC_50_ > 0.1), and, therefore, at clinically relevant plasma concentrations inhibition of OAT1 is likely, and DDI with OAT1 transporter substrates were defined as clinically relevant in our study. Next, cytotoxicity caused by all four antivirals was evaluated after exposure of ciPTEC for 24–72 h to the drugs. As a measure of cytotoxicity, cell viability was analyzed by cellular dehydrogenase capacity, metabolizing MTT into purple formazan. In the parent ciPTEC, viability was not affected by any of the antivirals (48 h, 1 mM), while adefovir, cidofovir, and tenofovir significantly affected cell viability in ciPTEC-OAT1, and only tenofovir slightly decreased ciPTEC-OAT3 viability (Fig. 5a). Antiviral-induced toxicity was evaluated in more detail, demonstrating a concentration- and time-dependent decrease in viability by adefovir, cidofovir, and tenofovir in ciPTEC-OAT1, while the effect was less pronounced in ciPTEC-OAT3 (Fig. 5b and Table IV). These findings indicate the direct involvement of the OAT transporters in antiviral-mediated nephrotoxicity, although IC_50_ values found in the current study are higher compared to those obtained in previous studies (Table IV). The cytotoxic effect of the antivirals correlated nicely with the inhibitory effect on fluorescein uptake, except for zidovudine. Despite a clear inhibition of fluorescein uptake by zidovudine, suggesting OAT-mediated uptake, this compound did not affect cell viability as determined by the MTT assay. To investigate a potential protective effect via intact efflux transporters in ciPTEC, cells were exposed to zidovudine at 10× C_max_ (50 μM) in presence of MRP4 and BCRP inhibitors MK571 and KO143, respectively. This did not affect cell viability in ciPTEC, ciPTEC-OAT1, nor ciPTEC-OAT3, indicating that efflux transporters did not counteract intracellular exposure of zidovudine and thereby reducing the cytotoxic potential of zidovudine.

**Table III:** Inhibitory Potencies of Antivirals on Fluorescein Uptake Using ciPTEC-OAT1 and ciPTEC-OAT3 Compared with a Selection of Reference Values. In the Current Study, Fluorescein Inhibition by the Model Compounds was Measured. For References, the Competitive Substrate is Provided

	Current study		Literature					DDI index		
	Cell line	IC_50_ (μM)	IC_50_ (μM)	K_m_ (μM)	Substrate	Cell line	Ref.	C_max_ (μM)	C_max_/IC_50_	Ref
Adefovir	ciPTEC-OAT1	23 ± 4	8.1 28	23.8	PAH 6-carboxyfluorescein –	HeLa-OAT1 CHO-OAT1 CHO-OAT1	(27,39)	1.6 38.8	0.18 4.2	(40,41)
	ciPTEC-OAT3	N.A.								
Cidofovir	ciPTEC-OAT1	71 ± 34	60	58	6-carboxyfluorescein –	CHO-OAT1 CHO-OAT1	(27)	15.8 26.3	0.53 0.88	(42,43)
	ciPTEC-OAT3	N.A.								
Tenofovir	ciPTEC-OAT1	42 ± 8	29.3	33.8	PAH –	HeLa-OAT1	(39,44)	0.52 0.72	0.014 0.019	(45,46)
	ciPTEC-OAT3	N.A.								
Zidovudine	ciPTEC-OAT1	14 ± 7		45.9	–	S2-OAT1	(16)	5.5	0.55 0.66	(47)
	ciPTEC-OAT3	21 ± 4		145	–	S2-OAT1	(16)	6.6	0.69 0.83	(47)

*N.A* not applicable, *PAH* para-aminohippurate, *HeLa* human epitheloid cervix carcinoma cell, *CHO* Chinese hamster ovary cell line, *S2* SV40T immortalized mouse renal cell line

^a^Data are expressed as mean ± SEM

**Table IV:** Inhibitory Potencies of Antivirals on Cell Viability as Measured by MTT Assay Using ciPTEC-OAT1 and a Selection of Values as Found in Literature^a^

	ciPTEC-OAT1					
	Current study			Literature		
	24 h	48 h	72 h	48 h	120 h	Ref
Adefovir	462 ± 52	303 ± 38	230 ± 37	0.22 ± 0.08	1.4 ± 0.7	(44,49)
Cidofovir^b^	613 ± 384	130 ± 58	69 ± 2	0.5 ± 0.2	3 ± 1	(44,49)
Tenofovir	114 ± 25	189 ± 48	223 ± 67	10 ± 2	21 ± 7	(44,49)

^a^Data are expressed as μM (mean ± SEM), n ≥ 3.

^b^Apparent IC_50_ value due to partial inhibition

## DISCUSSION

To improve prediction of the nephrotoxic potential of novel chemical entities and to mechanistically understand the pathways associated with drug-induced toxicity, highly predictive and validated translational models are required. In the present report, we describe such a robust human-based cell model with intact proximal tubular characteristics. Stable OAT1 and OAT3 expression in the human renal cell line ciPTEC allowed studying reproducible DDI for a panel of model substrates and antiviral compounds. Functional OAT1 and OAT3 transport activity was demonstrated to be associated with drug-induced toxicity of the antivirals adefovir, cidofovir, and tenofovir. These findings indicate that our model predicts drug-induced nephrotoxicity and underscore that functional expression of influx transporters is pivotal in prediction of drug-induced renal toxicity.

Many reports related to studying drug-OAT interactions describe the use of non-polarized overexpression systems, such as Chinese hamster ovary (CHO) cells, the human cervical epitheloid carcinoma cell line HeLa, or human epithelial kidney (HEK) 293 cells, which are highly relevant for studying interactions at the single-transporter level but might have a poor overall predictivity due to their simplicity (27,39). Since proximal tubule cells are the main site of adverse drug effects in the kidney, this cell type is preferred for *in vitro* assays investigating drug-induced nephrotoxicity (1). Human primary proximal tubule cells reflect *in vivo* toxicological responses best, but lack reproducibility and robustness due to high donor-to-donor variability and limited availability. Moreover, primary cells lose their proximal tubular phenotype upon culturing, and OAT1-4, P-glycoprotein, and MRP expressions were found to be rapidly decreased (50,51). To extend the life span of human proximal tubular cells and to provide a robust model for drug screening, we and others have immortalized primary kidney cells, yet without demonstrating functional OATs (21,52), despite retained gene expressions (53).

The current study demonstrates the first human model with stable expression of OAT1 and OAT3 for up to 10 and 11 passages, respectively, as analyzed by qPCR and functionality of OAT1 and OAT3 for up to 29 passages as analyzed by fluorescein uptake. Experimental values obtained for DDI of model compounds correlated well with published data, confirming PAH has a higher inhibitory potency for OAT1 compared to OAT3, whereas the inhibitory potencies of estrone sulfate, probenecid, and furosemide were clearly higher for OAT3. The IC_50_ value of cimetidine in ciPTEC-OAT1 is, however, more than fivefold higher as described earlier, whereas ciPTEC-OAT3 inhibition by cimetidine was found well within predetermined ranges (34). This discrepancy may be explained by different substrates used in the studies, where the OAT1-substrate PAH used in earlier studies, has a lower affinity for OAT1 as compared to fluorescein used in the current study. Since tetracyclin-inducible expression of OAT1 and OAT3 in ciPTEC was not achieved, we hypothesize that random integration of the vector could have caused silencing of this particular promoter element. The effects of prototypic inhibitor compounds on drug transport are promising with respect to the application of ciPTEC as a tool to study drug-induced nephrotoxicity, and the proof-of-concept was evaluated further with a selected a panel of clinically relevant antivirals with various pharmacokinetic parameters.

DDIs are a major concern in anti-HIV therapy that includes co-administration of multiple antivirals. We evaluated adefovir, cidofovir, tenofovir, and zidovudine DDI at the site of OAT1 and OAT3. The affinities of adefovir, cidofovir, and tenofovir were higher for OAT1 than for OAT3, in agreement with previous studies in CHO cells overexpressing hOAT1 and hOAT3 (44). The DDI index has been used to determine the potential of clinical DDI and drug-induced toxicities (48,54) and allows extrapolating *in vitro* observations to the clinical setting (48,54). In our study, IC_50_ values of less than 10 times the maximal free plasma concentration (C_max,u_/IC_50_ > 0.1) were found for adefovir, cidofovir, and zidovudine, indicating these antivirals are likely to inhibit OAT1 and OAT3 at clinically relevant concentrations.

Antiviral-induced nephrotoxicity was shown to be associated with OAT-mediated uptake and further evaluated in the current study (11,15,44,49). We demonstrated that OAT1 or OAT3 expression is required for induction of toxicity by adefovir, cidofovir, and tenofovir in ciPTEC. The relation between OAT1 transporter affinity and toxicity was described earlier using HeLa cells, transiently expressing hOAT1, in which cidofovir showed a higher affinity as well as a higher toxicity compared to tenofovir (39). In agreement, when the cytotoxic potential of NtRTIs in ciPTEC-OAT1 at 72 h of exposure was ranked, we found that cidofovir has the highest potency over tenofovir and adefovir (44,49). On the other hand, the low potency of adefovir in our study contrasts to the cytotoxicity reported for other cell models (34,54). In general, the toxic potency of the antivirals in ciPTEC is lower as compared to hOAT1-CHO and HEK-OAT1, which may be due to the presence of functional metabolic enzymes and an intact efflux machinery in ciPTEC (44,49,55). RNA expression of phase I enzymes CYP3A4, CYP4A11, and several UDP-glucuronosyltransferases (UGTs) in ciPTEC were found to be comparable to their expression levels in primary PTEC (23,51). Protein expression of the efflux transporters Pgp and MRP4 was demonstrated, as well as functional efflux transport activity of Pgp, MRP4, and BCRP (21,24). From these findings, we conclude that ciPTEC closely reflects the physiological situation, suggesting that our model is of higher predictive value than single overexpression systems. Since MRP4 mediates the efflux of tenofovir, its functional presence in ciPTEC might explain the reduced cytotoxicity in our model as compared to overexpression systems lacking this transporter (15,55). Future research should clarify this.

Activity of phase I and phase II metabolizing enzymes was demonstrated in ciPTEC of which the UGT2B7 subfamily might have been the cause of the tolerance for zidovudine observed in the present study (23). While adefovir, cidofovir, and tenofovir are largely excreted unchanged by the kidneys, only 23% of zidovudine is eliminated via the urine without metabolic alterations (56). Zidovudine undergoes either phase II metabolism into the non-toxic 5’-zidovudine-O-glucuronide or the antiviral is phosphorylated resulting in mitochondrial toxicity (12,57). As both glucuronidation and phosphorylation take place at the same functional group of zidovudine (5’-OH), the low toxicity of zidovudine suggests a favor for glucuronidation in ciPTEC. Although glucuronidation predominantly takes place in the liver, UGT2B7 expression in ciPTEC might contribute to zidovudine detoxification. Moreover, the toxic side effects of nucleoside analogs have been correlated with the kinetics of incorporation by the mitochondrial DNA polymerase, ranking zidovudine less toxic than tenofovir (58). As efflux inhibition of MRP4 and BCRP did not further reduce viability of ciPTEC upon exposure with zidovudine, the toxicity of this compound is likely not influenced by these efflux transporters. Differences in expression of metabolic enzymes and transporter activities between various cell lines used for toxicity studies should be taken into account when comparing functional readout parameters. Moreover, the broad presence of metabolic enzymes and transporters in our model as well as in freshly isolated PTECs increases their predictive potential, but complicates comparison with more simple models. Taken together, the combined expression of efflux transporters (MRP4, BCRP, MATE2-K, and Pgp) with influx transporters (OAT1/3, OCT2, and SLCO4C1) and metabolic enzymes make ciPTEC suitable to study multiple steps involved in renal elimination and drug-induced nephrotoxicity.

The clinical relevance and impact on drug safety of OAT transporters are well acknowledged by regulatory authorities and the pharmaceutical industry (59). Both the FDA and the European Medicines Agency (EMA) have issued guidance documents, outlining that OAT interactions should be studied for new compounds (48,60). Furthermore, the International Transporter Consortium (ITC) provided decision trees to determine whether a drug candidate may be a substrate (victim) or an inhibitor (perpetrator) of transporters involved in clinically relevant DDI (61). Consequently, pharmaceutical industry started a quest for reliable and high-throughput *in vitro* models that mimic the human kidney with improved prediction of drug-induced nephrotoxicity and a decrease in use of animals in research (62). Current preclinical tests for prediction of nephrotoxicity are mainly based on animal (rodent) models. These models provide information about systemic toxicity in living organisms, but they bear high costs, are time intensive, and remain an ethical issue. Their clinical predictive value is limited due to inherent interspecies differences in drug disposition and emphasizes the urgent need for human-based models that closely resemble the human kidney physiology (6,63). Current innovations in *in vitro* models allowing cells to grow in polarized structures under flow conditions, in combination with high-throughput automated systems for toxicity read-outs, will become major steps forward in drug safety screening, for which the ciPTEC model may provide a suitable cellular basis (64). In general, application of ciPTEC as a predictive tool for drug-induced toxicity requires comparison with freshly isolated PTECs and further validation by extrapolation of *in vitro* data to clinical outcomes.

## CONCLUSION

We present the first human PTEC model with stable expression and functionality of OAT1 and OAT3, allowing screening for drug-induced nephrotoxicity and DDI. The NtRTI drugs tenofovir, adefovir, and cidofovir-induced nephrotoxicity and exhibited DDI indices at clinically relevant concentrations. These findings underscore that ciPTEC-OAT1 and ciPTEC-OAT3 are valuable tools for drug-induced toxicity screening that, upon systematic validation, could improve translation of *in vitro* findings to clinical research and might decrease the use of animal studies in the preclinical stages of drug development.

## Electronic Supplementary Material

Fig. S1 (PDF 14 kb)
