# Supplementary material for: A Human Renal Proximal Tubule Cell Line with Stable Organic Anion Transporter 1 and 3 Expression Predictive for Antiviral-Induced Toxicity
Source: AAPS J. 2016 Jan 28;18(2):465–75. doi: 10.1208/s12248-016-9871-8 (PMC4779111; doi:10.1208/s12248-016-9871-8)
Supplement: Supplementary file 1 — (PDF 14 kb) [file 12248_2016_9871_MOESM1_ESM.pdf]

A validated human renal proximal tubule cell line with stable organic anion transporter expression predictive for drug-induced toxicity

Tom TG Nieskens, Janny GP Peters, Marieke J Schreurs, Niels Smits, Rob Woestenenk, Katja Jansen, Thom K van der Made, Melanie Röring, Constanze Hilgendorf, Martijn J Wilmer and Rosalinde Masereeuw

Supplementary Information

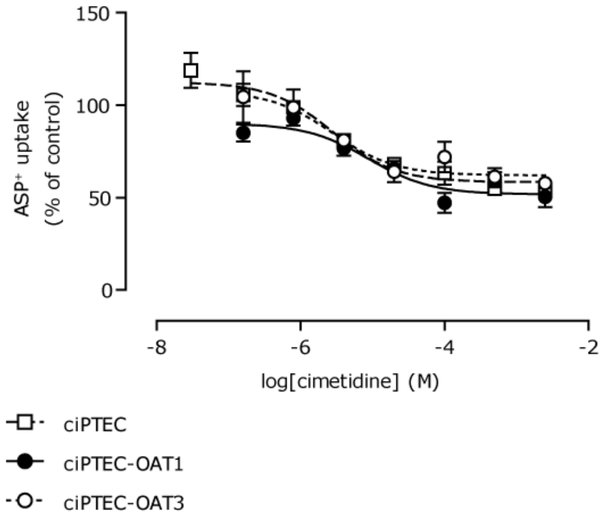

Supplementary Figure S1. Intact OCT2 transport after transduction procedures. ASP^+ uptake (1 µM) by ciPTEC parent, ciPTEC-OAT1 and ciPTEC-OAT3 when co-incubated with OCT2-substrate cimetidine for 60 min in HBSS at 37°C, relative to uptake without inhibitor. The lines represent the fit according to a one-site competition model. Values are expressed as ±SEM, (ciPTEC, n=3; ciPTEC-OAT1, n=4; ciPTEC-OAT3, n=2. Analysis using Two-way ANOVA indicated significant inhibition of ASP^+ uptake at OCT2 with cimetidine, resulted in similar IC_{50} ($p>0.05$).
